# Disappearing Integrative Motivation: A Validated Scale of Motivation for Learners of Southeast Asian Languages

**DOI:** 10.3390/bs15040464

**Published:** 2025-04-03

**Authors:** Xiaobin Ren, Lin Wang

**Affiliations:** School of Foreign Languages, Guangxi University, Nanning 530004, China; 2305301003@st.gxu.edu.cn

**Keywords:** learners of Southeast Asian languages, motivation scale, instrumental motivation, integrative motivation, thematic analysis

## Abstract

This study aimed to develop and validate a motivation scale specifically designed for university students enrolled in Southeast Asian language programs. Utilizing a thematic analysis of interviews with 28 students, five key motivational dimensions were identified: institutional environment, proficiency demand, self-development planning, social responsibilities, and intrinsic interest. These dimensions informed the construction of an initial scale, which was empirically tested and refined through two rounds of validation. The final 19-item scale covers four core dimensions, excluding intrinsic interest, reflecting the dominance of instrumental motivations in this context. Results highlighted the practical and goal-oriented nature of these motivations, differing from the integrative motivations observed in learners of global languages like English. This study fills a research gap by offering a validated tool for assessing language learning motivation in smaller, regionally significant language programs and provides insights into the unique motivational factors driving these learners.

## 1. Introduction

The study of foreign language learning motivation has long been recognized as a critical area in language acquisition research, as motivation profoundly shapes learners’ engagement (Alemayehu & Chen, 2023), persistence (Lee & Song, 2022), and ultimate success in achieving language proficiency (Tokan & Imakulata, 2019). With globalization driving closer economic, cultural, and political ties between nations, the study of Southeast Asian languages, such as Thai, Vietnamese, and Indonesian, is gaining international importance (Saddhono et al., 2024; Talodpob et al., 2025). As institutions worldwide increasingly offer programs in these languages (Hardini et al., 2023), understanding the specific motivational factors that drive students to learn them is essential. In China, for instance, universities have seen a growing emphasis on Southeast Asian language programs due to the country’s rapidly expanding relationships with the Association of Southeast Asian Nations (ASEAN) (Liu, 2023). In addition, the unique cultural challenges and strategic significance of Southeast Asian languages—central to fostering closer ties within the ASEAN region and beyond—make investigating the motivations of learners of Southeast Asian languages particularly relevant.

Over the past several decades, the study of foreign language learning motivation has gained considerable attention in the field of second language acquisition. Seminal frameworks, such as Gardner’s Socio-Educational Model (Gardner, 1988) and Dörnyei’s Motivational Self System (Dörnyei, 2014), have laid a foundation for understanding the complex factors that drive individuals to learn a second language. Much of the existing research, however, has focused predominantly on widely taught global languages, particularly English, and has explored the motivation of learners of Western languages, examining factors like integrative and instrumental motivation (Zhang et al., 2020), self-determination (Annamalai et al., 2023), and language learning anxiety (El Shazly, 2021). These investigations have largely been conducted within mainstream educational contexts, often overlooking learners of less commonly taught languages. For example, despite the extensive body of research on foreign language learning motivation, there remains a significant gap in understanding the unique motivational dynamics of students studying Southeast Asian languages. Learners of these languages are situated in a distinct linguistic and cultural context where motivation may be influenced by factors related to the geopolitical and economic importance of Southeast Asia in an increasingly interconnected world (Nikitina, 2022). However, the current literature does not fully explore the motivational profiles that influence these students’ engagement and persistence in learning Southeast Asian languages. Addressing this research gap is crucial, as it broadens the scope of motivation studies by incorporating a previously underexplored group of language learners, thereby providing a more nuanced understanding of how motivation functions across diverse linguistic and cultural settings.

The purpose of this study is to first explore the motivational dimensions of Southeast Asian language learners and, based on these findings, develop a reliable and valid measurement tool that accurately assesses the Southeast Asian language learning motivation of university students in China. To achieve this, we conducted semi-structured interviews with 28 Southeast Asian language majors and identified five key motivational dimensions through thematic analysis (Cooper et al., 2012). Based on these dimensions, we created a 22-item scale, which was tested and refined in two rounds with a large sample, resulting in a final 19-item scale that accurately measures the foreign language learning motivation of these students. The significance of this study lies in its development of a motivation scale specifically tailored to learners of Southeast Asian languages, filling a gap in language motivation research that has largely focused on widely taught languages like English. This scale provides a nuanced tool for understanding the unique motivational factors in this context, supporting more effective language learning strategies and educational programs.

## 2. Literature Review

### 2.1. Foreign Language Learning Motivation

Motivation plays a critical role in foreign language acquisition, with numerous studies confirming its influence on learners’ engagement (Alemayehu & Chen, 2023), persistence (Lee & Song, 2022), and ultimate success (Tokan & Imakulata, 2019) in language learning. Additionally, the relationship between foreign language learning motivation and other psychological factors, such as anxiety (Alrabai, 2024), self-efficacy (Prat-Sala & Redford, 2010), and willingness to communicate (Wu & Lin, 2014), has been explored extensively, with findings indicating that motivation significantly shapes many aspects of learners’ psychological and emotional states. These studies collectively emphasize the central role of motivation in the language learning process.

While significant progress has been made in understanding the relationship between motivation and foreign language learning for widely taught languages like English and Spanish, much less attention has been paid to learners of less commonly taught languages (Kong et al., 2018), despite their increasing relevance in global communication. For learners of less commonly taught languages, unique challenges arise due to limited resources, smaller learning communities, and fewer opportunities for interaction with native speakers (Kong et al., 2018; Torres & Turner, 2017; Yeh et al., 2015). Given these factors, existing motivation scales, most of which are designed for English language learners (Ardasheva et al., 2012; Yu et al., 2019), may not be suitable for assessing the motivation of learners of Southeast Asian languages. The distinct cultural, educational, and professional contexts in which these students operate require a tailored approach to measuring their motivation.

In recent years, motivation research on languages other than English (LOTE) has expanded, demonstrating significant variations across linguistic and cultural contexts. For instance, integrative motivation prominently drives Japanese learners in New Zealand (de Burgh-Hirabe, 2019), while instrumental goals, family influences, and cultural interests shape Chinese learners’ motivation to study Korean (Su et al., 2023). Similarly, studies on learners of multiple languages in Central Asia (Calafato, 2021) and Arabic language learners (Calafato, 2023) highlight the dynamic and context-dependent nature of language learning motivation. However, findings from these contexts may not directly apply to learners of Southeast Asian languages, given differences in economic, cultural, educational, and policy-related influences (Liddicoat, 2013). Thus, investigating Southeast Asian language learning motivation requires context-specific considerations, addressing a gap largely overlooked in existing research.

### 2.2. Southeast Asian Languages

Southeast Asian languages, including Thai, Vietnamese, and Indonesian, have gained geopolitical and economic importance due to the ongoing integration efforts within ASEAN aimed at creating a unified economic and sociocultural community (Korwatanasakul, 2022; Vo & Ho, 2025), as well as ASEAN’s growing role in global trade and diplomacy (Magno & Vivo, 2023). China’s expanding economic partnerships and diplomatic engagements with ASEAN countries, driven by strategic initiatives such as the Belt and Road Initiative (BRI) and the China–ASEAN Free Trade Area (Hong, 2019; L. Wang & Chen, 2025), have significantly increased the practical relevance and institutional support for these languages within Chinese higher education. Consequently, an increasing number of Chinese universities have introduced Southeast Asian language programs (Wong & Xun, 2019), reflecting heightened instrumental and institutional motivations among students to pursue proficiency in these languages. Therefore, an in-depth investigation into Southeast Asian languages as foreign languages is necessary, particularly focusing on exploring and analyzing the motivational factors driving university students to learn these languages. However, research on the teaching and learning of Southeast Asian languages remains limited, with most studies focusing on linguistic characteristics rather than language acquisition by non-native speakers. For example, Bisang (1996) explores grammaticalization processes in Southeast Asian languages, while Brunelle and Kirby (2016) examine tonal diversity and historical development in the region’s languages.

Although some research touches on Southeast Asian language education, these studies primarily address native speakers or heritage learners rather than foreign language learners. For instance, Chantarat and Chookhampaeng (2023) and Chanthao (2018) investigate Thai language instruction in Thai primary schools, focusing on challenges and approaches relevant to native Thai children. Similarly, studies on heritage language learning, such as Yeh et al. (2015) on Vietnamese in immigrant families in Taiwan and Jamcharoensup (2014) on Thai language instruction in Swiss Buddhist Sunday schools, emphasize cultural and familial dynamics in language retention rather than foreign language acquisition. While these studies provide valuable insights for native and heritage speakers of ASEAN languages, there is a lack of research on these languages as foreign languages, particularly focusing on learners in regions like China, where they are neither natively spoken nor maintained as heritage languages. Even fewer studies address the motivational factors that influence students learning these languages in foreign language contexts. This creates a significant gap in the literature, as learners studying Southeast Asian languages as foreign languages in China face unique challenges that differ considerably from those encountered by native or heritage speakers of these languages. For example, cultural differences between Southeast Asian countries and China are significant (Huang, 2019), influencing learners’ experiences and motivations in ways distinct from those learning them as native or heritage languages.

### 2.3. Integrative and Instrumental Motivation

Gardner and Lambert (1972) categorized foreign language learning motivation into two main types: integrative motivation and instrumental motivation. Integrative motivation refers to the desire to learn a language to connect with and potentially become part of the target language’s cultural community. In contrast, instrumental motivation is driven by practical factors, such as career advancement, academic success, or other tangible benefits. This framework has been widely utilized to analyze the motivational dynamics of language learners, especially in large-scale research on English as a foreign language, and has proven effective for understanding motivation in language learning contexts (Gardner, 2012; Zhang et al., 2020).

While these concepts have been thoroughly studied in the context of English, Spanish, and other widely taught languages, there is a notable gap in research that applies this framework to less commonly taught languages like Southeast Asian languages. Recent research has pointed to the need for context-specific motivation scales that reflect the diverse learning environments and linguistic challenges of less commonly taught languages (Li, 2023), including Southeast Asian languages. Despite this, the underlying principles of Gardner’s theory remain highly relevant, as learners of Southeast Asian languages may also be motivated by both integrative and instrumental factors, such as developing cross-cultural communication skills or capitalizing on economic opportunities within ASEAN countries.

By applying this framework to the study of learners of Southeast Asian languages, we aim to evaluate the final motivation scale specifically designed for learners of Southeast Asian languages, and to explore how the motivations of students in Southeast Asian language programs may differ from those learning widely taught languages, like English.

## 3. Questionnaire Design

### 3.1. Research Context

In recent years, there has been a growing interest in non-dominant languages, reflecting a shift towards greater linguistic diversity in language education worldwide. This trend is particularly evident in China, where Southeast Asian languages such as Thai, Vietnamese, Burmese, Khmer, Malay, and Indonesian have attracted increased attention due to the country’s strengthening economic, cultural, and diplomatic ties with ASEAN nations. As these connections deepen, more Chinese universities are establishing Southeast Asian language programs to meet the rising demand. Currently, 56 Chinese universities offer undergraduate programs in Southeast Asian languages, covering a total of 150 specialized academic programs. These programs enroll approximately 12,000 undergraduate students, indicating a significant number of learners engaging in the study of these languages. (These data come from the National College Admission Website: https://gaokao.chsi.com.cn/ (accessed on 6 March 2025), an official platform for university admissions in China, which is managed by the Ministry of Education of China.) These programs are especially prevalent in universities located in southwest China, where geographical proximity and historical connections to Southeast Asia support these academic offerings. Additionally, specialized foreign language universities, such as Beijing Foreign Studies University and Shanghai International Studies University, have also introduced Southeast Asian language programs, reflecting the strategic value of these languages for regional cooperation and engagement.

### 3.2. Participants

Purposive sampling (Campbell et al., 2020) was employed in this study for the selection of participants. To ensure that participants had sufficient familiarity with their respective Southeast Asian languages, only students who had completed at least three months of formal study in their programs were included. The participant selection was further guided by the following criteria: (1) representation across multiple universities to ensure institutional diversity, (2) selection from universities in geographically distinct regions, and (3) balance across language programs to prevent the over-representation of any single language group.

In the end, 28 undergraduate students from six universities across China were selected, representing four Southeast Asian language programs: Vietnamese, Thai, Burmese, and Indonesian. These universities encompass diverse regional and institutional contexts, including those in Southwest China, which share closer geographical and cultural ties with Southeast Asia, as well as specialized foreign language institutions that offer a broad range of language programs. The selected universities are located in five different cities, ensuring a diverse sample that captures multiple perspectives on Southeast Asian language learning. Detailed demographic and background information on the participants is provided in Table 1.

### 3.3. Data Collection and Analysis

Data for questionnaire design were collected through semi-structured, in-depth interviews. To facilitate the process, in-person interviews were conducted with students enrolled in Southeast Asian language programs at GXU and GXMZ, both located in NN, while students from universities in other cities participated in online interviews. The interviews were conducted by one of the two authors, who holds a PhD and is currently a faculty member at a school of foreign languages in a university located in a southwestern province of China. To ensure the semi-structured interviews effectively captured relevant information, an interview guide was developed prior to the interviews. This guide was reviewed by a PhD-holding teacher in the department of Southeast Asian languages, whose feedback led to refinements in the questions. The interview guide focused on exploring the factors influencing university students’ motivation to learn Southeast Asian languages. Example questions included “What do you think are some good practices at your university that positively impact your motivation to learn Thai/Vietnamese/Burmese/Indonesian language?” Before each interview, written informed consent was obtained, and participants granted permission for audio recording. The average interview period was approximately 39 min.

After conducting interviews with 28 participants, the audio recordings were transcribed into editable text. From these, 24 transcripts—approximately 85% of the total—were randomly selected for coding and analysis before data saturation was achieved; the remaining 4 transcripts, accounting for 15% of the total data, were used for the data saturation test (see Section 3.4). Each of the selected transcripts was imported into NVivo 14 software for line-by-line coding. Two coders independently analyzed the transcripts using a blind coding approach. Their coding results were then compared, and any significant discrepancies were resolved through discussion to ensure consistency and reliability.

Thematic analysis was performed in two main stages. In the first stage, initial codes were assigned to the raw interview data by carefully reading and identifying meaningful segments within the text, which were then labeled with descriptive concepts. This process yielded 79 initial codes, which captured the underlying meanings of the responses. In the second stage, these initial codes were further abstracted and consolidated into 22 sub-themes, representing broader, more conceptual categories that highlighted recurring patterns across the data. Table 2 provides an overview of the coding process and examples of key themes.

In the second stage of analysis, the identified sub-themes were further grouped into five overarching themes. These themes were more abstract, integrating various sub-themes into central concepts that aligned closely with the study’s objectives. This two-step coding process allowed for a comprehensive understanding of the motivational factors influencing learners of Southeast Asian languages by systematically refining the raw data into key themes. By organizing the data in this way, the analysis revealed the core dimensions of motivation among the participants, providing structured insights into their learning experiences. Table 3 demonstrates some of the coding processes used in this second stage.

### 3.4. Data Saturation Test

To ensure the saturation of our data, we conducted a coding check with the remaining four transcripts that were not included in the initial thematic analysis. An additional coder, who was not involved in the initial coding process, independently coded these four transcripts. We compared the coding results of these additional transcripts with the existing 24 analyzed ones. The review process indicated that no new sub-themes emerged from the last four transcripts, suggesting that our data had reached saturation. This implies that the collected data sufficiently covered the range of themes relevant to our study, and further interviews would likely not contribute additional insights.

## 4. Scale Design and Evaluation

### 4.1. Scale Design

The initial development of scale items was guided by the findings from the thematic analysis of qualitative interview data. Through this analysis, we identified five key motivational dimensions: institutional environment, proficiency demand, self-development planning, intrinsic interest, and social responsibilities. Collectively, these dimensions encompassed a total of 22 sub-themes, representing specific motivational factors reported by participants.

To ensure that the scale items accurately reflected the real-world experiences of Southeast Asian language learners, we examined the original interview transcripts and derived 22 preliminary items, each corresponding to one of the identified sub-themes. These items were carefully worded to align with the participants’ own expressions while maintaining conceptual clarity.

### 4.2. Content Validity Test

After the initial development of the scale items, we conducted a content validity test by inviting two Southeast Asian language teachers to review the items. These two experts, who specialize in Vietnamese and Thai respectively, hold doctoral degrees in applied linguistics and have extensive experience in both foreign language teaching and research. The purpose of this review was to ensure that the items accurately reflected the characteristics of the measured dimensions, to identify any redundant items, and to verify whether the items could achieve the intended measurement objectives. Based on the feedback from the two experts, we made several revisions, including the modification, addition, and deletion of certain items.

In addition, we invited two undergraduate students, one from the Vietnamese program and one from the Indonesian program, to pilot the questionnaire. They provided feedback on the questionnaire’s relevance, readability, comprehensibility, and formatting. Their suggestions were incorporated, leading to further adjustments where necessary.

As a result, the final scale included two main sections. The first section gathered respondents’ background information, such as gender, university, major, and year of study. The second section consisted of 22 measurement items organized into five dimensions. These items were formatted using a 7-point Likert scale, where each question had 7 frequency options ranging from 1 to 7. Higher scores on this scale indicated stronger motivation for language learning.

### 4.3. Data Collection

The research team collected a total of 368 questionnaires from students enrolled in Southeast Asian language programs at various universities across China. These universities included foreign language universities, comprehensive universities, and ethnic universities, representing diverse regions such as Southwest China, East China, and North China. The diversity in both the types of universities and their geographical locations greatly enhances the representativeness of the collected data. After excluding questionnaires with missing data and responses from non-Southeast Asian language students, 308 valid questionnaires (Sample 1) were retained for analysis. The demographic information of these 308 respondents is presented in Table 4.

### 4.4. Data Analysis

To ensure the quality of the questionnaire, we first conducted item analysis and exploratory factor analysis (EFA) using data from 308 collected questionnaires. Both analyses were performed using SPSS 27 software.

#### 4.4.1. Item Analysis

To evaluate the reliability and stability of the initial questionnaire, we conducted item analysis using Cronbach’s alpha to assess the internal consistency of the entire scale. The overall Cronbach’s alpha value of the questionnaire was 0.907, indicating high reliability. Table 5 presents the Corrected Item–Total Correlation (CITC) for each item, as well as Cronbach’s alpha values for each dimension.

According to Sun et al. (2023), items with a CITC value below 0.4 should be removed from the scale to improve overall reliability. Based on this criterion, we deleted the items with CITC values lower than 0.4, including INI1 and INI3. Although the CITC value for item INI2 was greater than 0.4, we chose to remove it because Cronbach’s alpha value for the Intrinsic Interest dimension was only 0.484, which is below the recommended threshold of 0.7 for acceptable internal consistency (Taber, 2018). To improve the reliability of this dimension, we deleted all three items under the Intrinsic Interest dimension.

#### 4.4.2. Exploratory Factor Analysis

To examine the construct validity of the scale, an exploratory factor analysis was conducted after removing three items from the initial scale, leaving 19 items for analysis. Prior to performing the EFA, we conducted a Kaiser–Meyer–Olkin (KMO) test and Bartlett’s test of sphericity to ensure the data’s suitability for factor analysis. The KMO value was 0.892, which is above the recommended threshold of 0.5 (Kaiser, 1974), indicating that the sample was adequate for factor analysis. Bartlett’s test of sphericity yielded a chi-square value of 2733.838 (df = 171), which was statistically significant (*p* < 0.001), suggesting that the items were sufficiently correlated to proceed with the EFA (Bartlett, 1954).

We applied Principal Component Analysis as the factor extraction method and retained factors with eigenvalues greater than 1. Factor rotation was performed using the varimax rotation method. After rotation, the factor loadings for all items were inspected, and as shown in Table 6, all items had factor loadings greater than or equal to 0.5, indicating strong associations with the extracted factors (Chen & Tsai, 2007). Based on the factor loadings and theoretical considerations, we retained all 19 items. The final scale consisted of four factors, accounting for 63.65% of the total variance.

## 5. Final Scale Validation and Evaluation

### 5.1. Data Collection

To further validate and evaluate the final version of the scale, we conducted a second round of data collection. In this round, we distributed the revised questionnaire (with 19 items) to a new sample of participants from different universities than those used in the first round. Specifically, we expanded our sample to include universities in the South China region, which had not been covered in the first round of data collection. This approach ensured greater geographic and institutional diversity in our sample, enhancing the generalizability of the findings. A total of 523 questionnaires were collected during this second phase. After removing incomplete responses and responses from participants whose academic backgrounds did not meet the study’s criteria, we obtained 481 valid questionnaires (Sample 2). The demographic and academic information of the 481 participants is presented in Table 7. This table provides details on their institutional affiliations, academic fields, gender, and other relevant background variables. The inclusion of participants from a broader range of universities, particularly from South China, allows us to test the scale across different educational settings, thereby contributing to the robustness and validity of the scale’s application in diverse contexts.

### 5.2. Data Analysis

#### 5.2.1. Reliability Retest and Validity Analysis

To further ensure the reliability and validity of the revised scale, we conducted a retest using the data collected in the second round of data collection through SPSS 27. The results demonstrated that the overall Cronbach’s alpha coefficient for the entire questionnaire was 0.913, indicating a high level of internal consistency. Additionally, the reliability scores for each of the four dimensions were all above 0.7 (see Table 8), which further confirms the strong reliability and stability of the questionnaire.

In terms of validity, we examined the composite reliability (CR) and average variance extracted (AVE) for each dimension. The results showed that all dimensions had CR > 0.7 and AVE values greater than 0.5 (see Table 8), indicating that the revised questionnaire met the requirements for convergent validity (Fornell & Larcker, 1981).

Furthermore, as shown in Table 9, the square root of the AVE for each of the four factors on the diagonal was greater than the correlation coefficients between the factors, indicating that the scale demonstrated adequate discriminant validity. This suggests that the factors measured by the scale were sufficiently distinct from one another, further supporting the validity of the scale (Fornell & Larcker, 1981).

The diagonal values represent the square root of AVE for each dimension, indicating their own explained variance. The off-diagonal values are the correlation coefficients between dimensions, demonstrating that the square root of AVE for each factor is greater than its correlations with other factors, thus confirming discriminant validity.

#### 5.2.2. Confirmatory Factor Analysis

**Figure 1:**
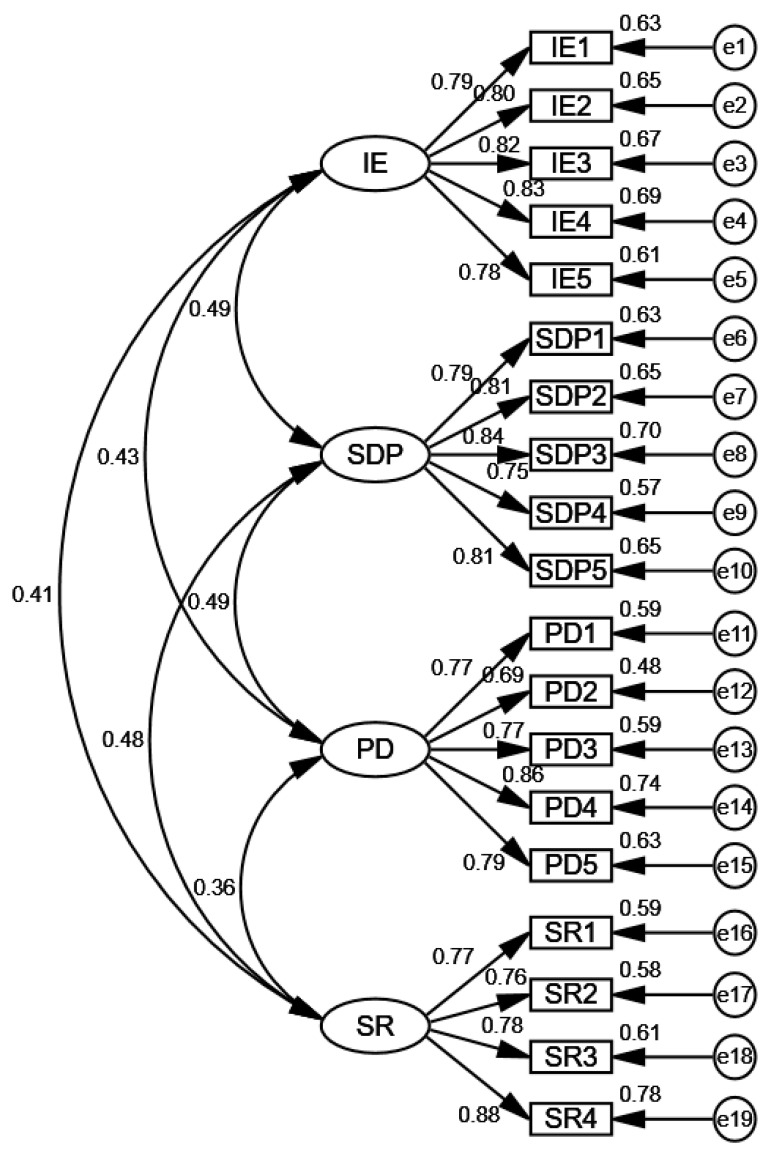
Structural equation model of learning motivation.

To further examine the stability of the four-factor structure of the scale, we conducted a confirmatory factor analysis (CFA) using AMOS 22 software on Sample 2. This analysis used the 4 factors identified from the exploratory factor analysis as latent variables, with the corresponding 19 items serving as observed variables. A structural equation model was constructed, with the results presented in Figure 1 and Table 10.

The CFA results indicated that all standardized factor loadings for the items were above 0.6, showing strong associations between the items and their respective latent factors. Additionally, the fit indices in Table 10 confirmed that the model fit was acceptable. The indices, including chi-square/df, CFI, GFI, RMSEA, and SRMR, all met acceptable thresholds for model fit. Therefore, the final version of the scale demonstrates strong psychometric properties and provides a valid measurement framework for assessing foreign language learning motivation among university students in Southeast Asian language programs.

## 6. Discussion

This study identified five key motivational dimensions in Southeast Asian language learning: institutional environment, proficiency demand, self-development planning, social responsibilities, and intrinsic interest. However, during the subsequent scale development and validation process, we found that intrinsic interest was not a significant factor influencing foreign language learning motivation in this context.

### 6.1. The Limited Role of Integrative Motivation

While thematic analysis initially revealed a dimension related to integrative motivation—captured as intrinsic interest—the large-scale validation of our scale did not confirm this dimension as a significant factor for students learning Southeast Asian languages. One key reason for this discrepancy lies in the methodological differences between qualitative and quantitative research. Qualitative thematic analysis captures a wide range of learner experiences, even those that are not widely shared, whereas quantitative validation requires high internal consistency across a large sample (Fugard & Potts, 2015). The low CITC values for the intrinsic interest items suggest that this factor was not consistently shared among most learners, making it unsuitable for inclusion as a core dimension in the final scale. Additionally, thematic analysis relies on a smaller, more diverse set of interviewees, where individual differences can strongly influence emerging themes. While some students in the qualitative phase expressed cultural curiosity, the large-scale survey, which offers a more representative picture, indicated that this motivation is not prevalent enough to form a stable factor. Moreover, intrinsic interest likely functions as a secondary rather than a primary motivational factor, as its weak association with other dimensions suggests it does not play a central role in driving language learning.

This result contrasts with findings from numerous studies on English as a foreign language, where integrative motivation consistently emerges as a prominent influence. For example, research by Gardner (2012) and Zhang et al. (2020) found that learners of English frequently cite the desire to integrate with English-speaking cultures as a core motivation, which strongly influences their engagement and persistence in learning the language.

One possible explanation for the weak integrative motivation among learners of Southeast Asian languages lies in the program placement process: many students enrolled in Southeast Asian language programs were assigned to these majors through a placement or “adjustment” process after not being admitted to their first-choice program (B. Wang, 2021). Several interviewees explicitly mentioned that they did not choose their current major voluntarily but were instead assigned to it by the university, which led to initial frustration and disengagement. For instance, one participant stated “I originally applied for Economics, but I was placed into the Thai program. At first, I had no interest in learning Thai at all.” This assignment often results in students studying a language they had not intended to learn and, in some cases, one they had not previously considered. This lack of initial personal interest can dampen their motivation to integrate with the target language culture, as their enrollment is not driven by a personal desire for cultural or linguistic connection.

Another important factor affecting integrative motivation among students of Southeast Asian languages may be related to power dynamics and perceived status. Compared to English-speaking countries, which are often associated with wealth, global influence, and cultural dominance (Pennycook, 2017), Southeast Asian nations generally hold a lower position in terms of economic and social power on the global stage. This perception was reflected in our interviews, where some participants explicitly stated that they felt learning a Southeast Asian language offered fewer opportunities for career advancement compared to English. One interviewee noted “When I tell people I study Burmese, they often ask why I didn’t choose a more ‘useful’ language like English or French.” As Norton’s Theory of Investment (Norton, 1995) suggests, learners are often drawn to languages linked with high-power, high-status societies where integration offers substantial social or economic advantages. However, Chinese students may not perceive the same incentive to integrate into Southeast Asian cultural communities, which are seen as less influential compared to Western, English-speaking countries. This dynamic results in weaker integrative motivation, as the prospect of connecting with Southeast Asian cultures may not carry the same allure or prestige.

Media representation further compounds this issue. In Chinese media, Western countries, especially English-speaking ones, receive extensive coverage, often portrayed as economically advanced and culturally rich (Xiong & Zheng, 2022). To some extent, this also reflects the media imperialism of Western countries (Chadha & Kavoori, 2000). In contrast, media coverage of Southeast Asian countries tends to be limited and, when present, sometimes focuses on challenges or negative aspects (Bao & Yuan, 2023). This perception was echoed in our interviews, where several participants mentioned that their exposure to Southeast Asian cultures was minimal before enrolling in their programs. One interviewee stated, “Most of what I knew about Vietnam before learning the language came from historical war documentaries rather than contemporary media”. This skewed portrayal reduces the cultural appeal of Southeast Asian countries for Chinese learners, thereby diminishing the sense of personal or cultural connection that integrative motivation typically requires.

Together, these methodological and sociocultural factors suggest that Chinese students may lack the integrative drive to immerse themselves in Southeast Asian cultures. While qualitative analysis revealed intrinsic interest among some learners, large-scale validation demonstrated that this motivation is neither consistently influential nor statistically robust. This reality, rooted in both research methodology and social factors, sets the stage for instrumental motivation to become the primary driver in learning these languages, as students focus on practical benefits rather than cultural integration.

### 6.2. The Predominance of Instrumental Motivation

Our study revealed that institutional environment, proficiency demand, self-development planning, and social responsibilities are the primary factors driving motivation among students in Southeast Asian language programs. These factors align closely with the concept of instrumental motivation, which emphasizes practical and goal-oriented reasons for language learning, such as career advancement, meeting academic requirements, and fulfilling social responsibilities. Similar findings have been observed in research on widely taught foreign languages like English. For instance, studies on English language learners highlight the importance of institutional support, skill proficiency goals, career planning, and societal roles as key motivators (Gardner & MacIntyre, 1991). In addition, many other studies have found that people’s motivation to learn LOTE, such as Arabic (Alhamami & Almosa, 2023), Spanish (Zheng et al., 2019), and Japanese (Z. Wang & Zheng, 2021), was also primarily driven by instrumental motivation. This alignment suggests that, across different language contexts, instrumental motivations share core components that drive learners toward tangible achievements. While the foundational aspects of instrumental motivation appear consistent with previous research on languages like English and LOTE, the unique contribution of this study lies in its focus on Southeast Asian languages. By examining instrumental motivation within the context of Southeast Asian languages learners in China, this study contributes to filling the gap in motivational research on smaller, regionally important languages.

While instrumental motivation has been widely studied in the context of English and LOTE, the specific drivers of motivation for Southeast Asian languages require closer examination due to the distinct socio-economic and cultural factors influencing learners’ decisions. For example, unlike learners of English who often pursue the language for global mobility (Lasanowski, 2011) and international job opportunities (Akther, 2022), students in Southeast Asian language programs in China tend to be more motivated by factors such as ASEAN job markets, China’s increasing economic integration with Southeast Asia, and governmental initiatives like the Belt and Road Initiative. Familial expectations also play a crucial role, with some students reporting that their parents encouraged them to study these languages due to perceived career advantages in regional trade and diplomacy.

Interestingly, one dimension commonly associated with instrumental motivation to learn English—informational media motivation—did not emerge in this study. Previous research has shown that English learners often view language proficiency as a means to access advanced scientific knowledge, cutting-edge technology, and international communication (Duff, 2017; Ushioda, 2017). As English serves as the global lingua franca, learning English provides direct access to a vast body of information, international resources, and leading scientific developments. However, Southeast Asian countries, while culturally and economically significant, generally lack the same level of influence in scientific and technological domains compared to English-speaking countries. Therefore, for students in China learning Southeast Asian languages, the motivation to access global information through language learning is less pronounced. This distinction underscores the importance of context in understanding instrumental motivation, suggesting that learners’ motivations are deeply shaped by the global and regional roles of the languages they study.

## 7. Conclusions

This study developed and validated a motivation scale tailored specifically for China’s university students in Southeast Asian language programs, addressing the distinct motivational factors influencing this unique learner population. Initially, we conducted thematic analysis of interviews with 28 students, identifying five motivational dimensions: institutional environment, proficiency demand, self-development planning, social responsibilities, and intrinsic interest. These dimensions provided a foundational framework for constructing the initial scale. Based on this framework, we designed and tested a series of scale items, conducting two rounds of empirical testing and validation. The final scale, consisting of 19 items across four core dimensions—excluding intrinsic interest—offers a reliable tool for assessing the specific motivations of learners of Southeast Asian languages in China.

The findings underscore the dominance of instrumental motivations in this context, highlighting practical and goal-oriented factors such as institutional support, proficiency requirements, career advancement, and social responsibilities as the primary drivers of motivation. This instrumental emphasis reflects a notable departure from the integrative motivations often found in studies of major world languages like English. The exclusion of intrinsic or integrative motives, such as a desire to culturally integrate with Southeast Asian communities, suggests that Chinese students in this field are largely motivated by practical outcomes, likely shaped by the socio-political and economic contexts surrounding these languages.

The significance of this study lies in its contribution to language motivation research by addressing a critical gap. Previous studies have primarily focused on widely taught global languages, often overlooking the unique motivations of learners of smaller, regionally significant languages. This study not only provides a robust scale for assessing motivation in Southeast Asian language programs but also broadens our understanding of instrumental and integrative motivations in language learning.

## 8. Limitations and Future Research

While our findings suggest that intrinsic interest did not emerge as a core motivational dimension in the large-scale validation, the qualitative phase indicated that certain students expressed intrinsic motivation linked to cultural and linguistic curiosity. Future research could explore alternative ways to measure intrinsic motivation, such as refining and expanding the intrinsic motivation dimension to enhance its reliability or developing a separate subscale specifically dedicated to intrinsic interest. Additionally, as Southeast Asian languages gain regional and global relevance, future studies may investigate how learners’ motivations evolve over time, particularly in relation to changing perceptions of these languages’ social and economic value. Furthermore, applying this motivation scale in other language learning contexts could provide comparative insights into the motivational dynamics of less commonly taught languages. Moreover, while our study focused on identifying key motivational dimensions, it did not examine how demographic factors such as geographic region, gender, academic year, language major, or institution type may influence motivation patterns. Given that variations in proximity to Southeast Asia, local economic opportunities, and institutional emphasis on Southeast Asian studies could shape learners’ motivations, future research could explore these factors in more depth.

## Figures and Tables

**Table 1:** Demographic information of the participants.

Name Code	Gender	Age	Grade	Major	University Code	Location Code
P1	M	22	4th	Vietnamese	GXU	NN
P2	F	21	2nd	Vietnamese	GXU	NN
P3	F	20	3rd	Thai	GXU	NN
P4	F	22	3rd	Burmese	GZU	GY
P5	F	19	1st	Burmese	GZU	GY
P6	F	20	3rd	Vietnamese	YNU	KM
P7	M	21	3rd	Vietnamese	YNU	KM
P8	F	23	4th	Thai	YNU	KM
P9	F	22	4th	Thai	YNU	KM
P10	F	20	3rd	Burmese	YNU	KM
P11	F	21	3rd	Burmese	YNU	KM
P12	F	18	1st	Vietnamese	HHC	HH
P13	M	19	1st	Thai	HHC	HH
P14	M	22	4th	Burmese	HHC	HH
P15	F	23	4th	Indonesian	TJFU	TJ
P16	F	22	3rd	Indonesian	TJFU	TJ
P17	F	23	4th	Indonesian	TJFU	TJ
P18	F	21	3rd	Thai	TJFU	TJ
P19	F	20	3rd	Thai	TJFU	TJ
P20	F	20	2nd	Burmese	TJFU	TJ
P21	F	21	2nd	Burmese	TJFU	TJ
P22	M	21	3rd	Indonesian	GXMZ	NN
P23	F	22	3rd	Indonesian	GXMZ	NN
P24	M	23	4th	Indonesian	GXMZ	NN
P25	F	21	2nd	Vietnamese	GXMZ	NN
P26	F	22	4th	Vietnamese	GXMZ	NN
P27	F	20	3rd	Thai	GXMZ	NN
P28	F	24	4th	Thai	GXMZ	NN

**Table 2:** Results of first coding stage.

Original Text	Initial Concepts	Sub-Themes
In some classes, students work well in groups. It is very interesting to discuss and learn together.	Collaboration enhances the fun of learning	Class environment
There was a teacher who was very knowledgeable, and I liked his teaching style.	Enjoy the teacher’s teaching style	Teachers’ competence
The tourist attractions in Thailand that the teacher talked about in class were very interesting.	The content of the lessons is interesting	Teaching contents
Our college has strict attendance policies, which urge me to focus on my studies.	Strict attendance supervision	University regulations
I believe these club activities are beneficial for learning Thai.	Club activities enhance learning motivation	Campus activities
Since I choose to learn this major, the most basic requirement is to get a graduation certificate.	Study for graduation	Graduation requirement
We had a teacher who was so strict with the class that I afraid of failing the final exam.	Study for exams	Pass examination
Now Vietnamese majors can also get the CATTI certificate. I’m going to study hard and try to get that certificate.	Study for certificates	Obtain certificate
I feel that the competition in our class is still quite fierce. So, if you want to get a scholarship, you need to take this course seriously.	Study for scholarships	Scholarship competition
Because if you want to get postgraduate recommendation, GPA is very important.	Study for postgraduate recommendation	Post-graduate entrance requirement
I am going to work for BYD Company in Thailand after graduation, so now I should work hard to improve my Thai language skills.	Need language skills to work abroad	Career planning
I plan to go to an Indonesian university for a master’s degree after graduation.	Study abroad requires language skills	Further study abroad
We will go to Vietnam for exchange study in the junior year, so I need to learn Vietnamese to prepare for it.	Exchange program needs language foundation	Student exchanges
When I travel to Indonesia, I should speak some Indonesian while experiencing the local customs.	To experience culture abroad	Travel abroad
There are a lot of Burmese students in the school, and I want to have more contact with them.	Needs to contact with international students	Communication needs
Maybe it’s because I’m personally interested in Vietnamese.	Interested in Vietnamese	Target language interest
When I was in Thailand, if I encountered festivals such as the Songkran Festival, I would definitely go out to play, because I wanted to experience the local customs.	Interested in Thai culture and festivals	Target culture interest
I have been good at language learning since I was a child, and I think I am also good at English.	Good at language learning	Aptitude in language learning
I was influenced by my uncle. My uncle went to Indonesia right after he graduated from college.	Influence of family members	Relatives’ expectation
If the relationship between China and Vietnam is good, there will certainly be more opportunities and better prospects for development.	Bilateral relations between countries influence learning expectations	China–ASEAN relations
Learning Burmese well can make more contributions to the economic, trade and cultural exchanges between China and Myanmar.	Economic ties promote language learning	Economic cultural ties
I think learning Southeast Asian languages well can help China increase its influence in ASEAN.	To enhance international influence	Political mission

**Table 3:** Results of second coding stage.

Sub-Themes	Themes	Connotation of Themes
Class environment	Institutional environment	The university’s resources, support systems, and policies influence students’ motivation to pursue language studies.
Teachers’ competence		
Teaching contents		
University regulations		
Campus activities		
Graduation requirement	Proficiency demand	Academic requirements drive students to achieve proficiency in their target language.
Pass examination		
Obtain certificate		
Scholarship competition		
Post-graduate entrance requirement		
Career planning	Self-development planning	Language skills are viewed as essential for students’ personal growth and future opportunities abroad.
Further study abroad		
Student exchanges		
Travel abroad		
Communication needs		
Target language interest	Intrinsic interest	A genuine passion for the language and culture sustains students’ motivation.
Target culture interest		
Aptitude in language learning		
Relatives’ expectation	Social responsibilities	Students feel a duty to meet family expectations and support national economic and political interests through language learning.
China–ASEAN relations		
Economic cultural ties		
Political mission		

**Table 4:** Characteristics of Sample 1.

Feature	Category	Frequency	Proportion/%
Gender	Male	46	14.94
	Female	262	85.06
University type	Foreign language universities	61	19.81
	Comprehensive universities	163	52.92
	Ethnic universities	84	27.27
University location	Southwest China	159	51.62
	East China	70	22.73
	North China	79	25.65
Grade	1st Year	69	22.40
	2nd Year	77	25.00
	3rd Year	89	28.90
	4th Year	70	22.73
	other	3	0.97

**Table 5:** Cronbach’s Alpha and CITC for each dimension and items.

Dimensions	Items	Item Content	CITC	Cronbach α
Institutional environment	IE1	I enjoy the collaborative learning environment in my classes.	0.497	0.845
	IE2	I appreciate the teaching methods used by my teachers.	0.583	
	IE3	The content of the courses is helpful and engaging.	0.527	
	IE4	The university’s rules and regulations motivate me to study harder.	0.547	
	IE5	Campus activities enhance my learning motivation.	0.537	
Proficiency demand	PD1	I study hard to meet the graduation requirements.	0.523	0.816
	PD2	Passing exams is important to prove my abilities.	0.496	
	PD3	Obtaining a certificate is a key goal for me.	0.608	
	PD4	I strive to win scholarships, which drives me to study diligently.	0.598	
	PD5	The entrance requirements for graduate school encourage me to improve academically.	0.503	
Self-development planning	SDP1	My career goals push me to learn the target language.	0.547	0.848
	SDP2	Studying abroad is part of my plan for self-improvement.	0.609	
	SDP3	I am preparing to participate in student exchange programs.	0.637	
	SDP4	I want to travel abroad to enhance my cultural understanding.	0.588	
	SDP5	Communicating with international students motivates me to improve my language skills.	0.621	
Social responsibilities	SR1	My family expects me to do well in learning the target language.	0.564	0.843
	SR2	I want to contribute to China-ASEAN relations through language learning.	0.509	
	SR3	Economic and cultural ties between countries inspire me to learn the language.	0.477	
	SR4	I feel that learning this language will help enhance China’s global image	0.549	
Intrinsic interest	INI1	I am genuinely interested in learning the target language.	0.338	0.484
	INI2	I am curious about the culture of the target language country.	0.495	
	INI3	I believe I have a natural talent for learning languages.	0.356	

**Table 6:** Factor loadings of the items.

Items	Factors			
	IE	PD	SDP	SR
IE1	0.59			
IE2	0.66			
IE3	0.60			
IE4	0.63			
IE5	0.59			
PD1		0.60		
PD2		0.54		
PD3		0.66		
PD4		0.65		
PD5		0.58		
SDP1			0.62	
SDP2			0.67	
SDP3			0.70	
SDP4			0.65	
SDP5			0.68	
SR1				0.61
SR2				0.52
SR3				0.50
SR4				0.59

Note: IE = institutional environment; PD = proficiency demand; SDP = self-development planning; SR = social responsibilities.

**Table 7:** Characteristics of Sample 2.

Feature	Category	Frequency	Proportion/%
Gender	Male	95	19.75
	Female	386	80.25
University type	Foreign language universities	155	32.22
	Comprehensive universities	213	44.28
	Ethnic universities	113	23.49
University location	Southwest China	112	23.28
	East China	106	22.04
	North China	125	25.99
	South China	138	28.69
Grade	1st Year	51	10.60
	2nd Year	97	20.17
	3rd Year	186	38.67
	4th Year	144	29.94
	other	3	0.62

**Table 8:** Reliability and convergent validity of the questionnaire.

Dimension	Cronbach’s Alpha	Composite Reliability	AVE
Institutional environment	0.900	0.902	0.649
Proficiency demand	0.874	0.884	0.605
Self-development planning	0.897	0.899	0.640
Social responsibilities	0.882	0.876	0.639

**Table 9:** Discriminant validity of the questionnaire.

	AVE	IE	PD	SDP	SR
IE	0.649	0.806			
PD	0.605	0.425	0.778		
SDP	0.640	0.495	0.490	0.800	
SR	0.639	0.406	0.358	0.479	0.799

Note: IE = institutional environment; PD = proficiency demand; SDP = self-development planning; SR = social responsibilities.

**Table 10:** Model fit indices.

Fit Index	Result	Fit Criterion
χ^2^/df	2.041	<3.0 (Kline, 2023)
GFI	0.938	>0.90 (Kline, 2023)
AGFI	0.919	>0.90 (Kline, 2023)
CFI	0.972	>0.90 (Kline, 2023)
TLI	0.967	>0.90 (Schermelleh-Engel et al., 2003)
IFI	0.972	>0.90 (Schermelleh-Engel et al., 2003)
RMSEA	0.047	<0.08 (Kenny et al., 2014; Schermelleh-Engel et al., 2003)
SRMR	0.035	<0.10 (Schermelleh-Engel et al., 2003)

## Data Availability

Data are contained within the article.
